# Effectiveness of a new long-lasting insecticidal nets delivery model in two rural districts of Mozambique: a before–after study

**DOI:** 10.1186/s12936-018-2217-5

**Published:** 2018-02-05

**Authors:** Jorge A. H. Arroz, Baltazar Candrinho, Chandana Mendis, Pablo Varela, João Pinto, Maria do Rosário O. Martins

**Affiliations:** 1 World Vision International, Maputo, Mozambique; 2 National Malaria Control Programme, Maputo, Mozambique; 3 Global Health and Tropical Medicine, GHTM, Instituto de Higiene e Medicina Tropical, IHMT, Universidade Nova de Lisboa, UNL, Rua da Junqueira 100, 1349-008 Lisbon, Portugal

**Keywords:** Long-lasting insecticidal nets campaign, Universal coverage, New and old delivery model, Before-after study, Mozambique

## Abstract

**Background:**

In 2015, Mozambique piloted a new model of long-lasting insecticidal nets (LLINs) delivery in a campaign. The new delivery model was used in two rural districts were, and two others were considered as control, maintaining the old delivery model. The aim of this study is to compare the coverage of ownership and use of LLINs in intervention and control districts in Mozambique.

**Methods:**

A before-after design with control group was carried out 6 months after LLINs distribution. Using systematic probabilistic sampling, 1547 households were surveyed by means of a questionnaire. To find associations between the district categories (intervention and control) and the main outcomes of the study (LLIN ownership, use, and universal coverage achievement), odds ratio (OR) and respective confidence intervals were calculated.

**Results:**

Of the 760 households surveyed in the intervention districts, 98.8% had at least one LLIN; of the 787 households surveyed in the control districts, 89.6% had at least one LLIN [OR: 9.7, 95% (CI 4.84–19.46)]. Around 95 and 87% of households owning at least one LLIN reported having slept under the LLIN the previous night in the intervention and control districts, respectively [OR: 3.2; 95% (CI 2.12–4.69)]. Seventy-one percent of the households surveyed achieved universal coverage in the intervention districts against 59.6% in the control districts [OR: 1.6; 95% (CI 1.33–2.03)].

**Conclusions:**

The universal coverage campaign piloted with the new delivery model has increased LLINs ownership, use, and progression for reaching universal coverage targets in the community.

## Background

Using long-lasting insecticidal nets (LLINs) can reduce malaria morbidity and mortality, especially in children and pregnant women [1, 2], and the universal coverage LLIN campaign is a proven health intervention toward this goal [3–5]. For countries in sub-Saharan Africa, it is estimated that 60% of at-risk population for malaria infection had access to an LLIN in 2015 and an estimated 53% of the population at risk slept under an LLIN in 2015 [6]. Ownership and use of LLINs in Mozambique increased between 2011 and 2015, but remain far from the desired targets. Households with at least one LLIN increased from 51% in 2011 to 66% in 2015; the mean LLINs per household increased from 0.9 in 2011 to 1.5 in 2015; the use of LLINs amongst children’s under five increased from 35.7% in 2011 to 47.9% in 2015; the universal coverage goal (one LLIN for every two persons) is still low, with 38.9% of households achieving this target in 2015 against 22.6% in 2011 [7, 8].

Between October and December 2015, Mozambique piloted a new model of LLIN delivery in an intervention campaign. The new LLIN delivery model was used in two rural districts (Gurue and Sussundenga), and two (Alto Molocue and Machaze) were considered as control maintaining the “old” delivery model [9].

Household registration in the control districts (“old” delivery model) was carried out by collecting variables such as name, age, gender, and family relationship of household members, and later analysed regarding possible sleeping patterns. Users’ sleeping patterns were the LLIN allocation criteria in the “old” delivery model. Household registration in the intervention districts (new delivery model) was carried out by collecting the number of household members, attributing a coupon to each registered household, and issuing an identification sticker to the household. The number of LLINs allocated to each household was obtained by dividing the number of household members by two (observing the principle of one LLIN for every two persons, rounded up to the next whole number) [9].

The aim of this study is to compare the coverage of ownership and use of LLINs in the intervention and control districts in Mozambique. The specific objectives are: (i) to estimate LLIN ownership amongst households in the intervention and control districts; (ii) to estimate LLIN use coverage in the intervention and control districts; (iii) to estimate the proportion of households reaching universal coverage target (one LLIN for every two person) in the intervention and control districts; and iv) to compare the ownership and use of LLINs in the intervention and control districts.

## Methods

### Context

The study was conducted in four districts: Gurue, Alto-Molocue, Sussundenga, and Machaze. The districts of Gurue and Alto-Molocue are located in the province of Zambezia and have estimated populations of 403,558 and 375,504 inhabitants in 2015, respectively [10]. The districts of Sussundenga and Machaze are located in the province of Manica and have estimated populations of 165,616 and 134,515 inhabitants in 2015, respectively [10]. All four districts are rural type, with hardship health services access, and low social and economic conditions. In 2015 malaria prevalence in Zambezia and Manica was 67.9 and 25.5%, respectively [8].

### Study design

A before-after design with control group was carried out 6 months after LLINs distribution, i.e., between June and July 2016. Two groups were considered: intervention (districts of Gurue and Sussundenga) and control (districts of Alto-Molocue and Machaze). These districts were selected based on the following matching criteria: (i) population size similarities; (ii) geographical area; (iii) similarity in the number of LLINs allocated for distribution; and (iv) having rural characteristics [9].

All the localities of these districts were selected for the survey. Within each locality household sample size was calculated by dividing the total sample size of the district by the number of existing localities. After determining the number of households in each locality, households were selected using systematic probabilistic sampling method. For both intervention and control districts the following household definition was assumed: includes all the people who live together or sleep in the same house/yard/plot and share the same food at meal times. When a man has more than one wife or woman, each of them is considered as a separate household.

### Study sample size

For each group, sample size was computed in order to detect a significant difference of 10% between the intervention and control: p1 (intervention) = 80%; p2 (control) = 70%; alpha = 0.05; power = 0.9; C_*p*,*power*_ = 10.5. Therefore, the sample size for each group was 776 households, i.e., each district had 388 households as sample size.

$$n = \frac{{[p_{1} (1 - p_{1} ) + p_{2} (1 - p_{2} )]}}{{(p_{1} - p_{2} )^{2} }} \times c_{{{\text{p}},\,{\text{power}}}}.$$

### Sampling strategy

A systematic random sampling was used in which every Nth member of the target population is selected to be included in the study. The sampling unit is the household.

### Selection of households

In each locality, the households were selected based on the following strategy: first, households list (population frame) was identified and a number assigned to each household; then, the sample interval (number of households divided by sample size) was computed and a random number was chosen to start with; finally, from this first random number, households were systematically selected until the sample size was complete.

### Data collection

A semi-structured questionnaire with open and closed questions was used. Before the beginning of the study a pilot study took place by applying the questionnaire to 20 households located in districts that were not part of the study. Some adjustments were made to improve the original version of the questionnaire. In order to avoid information bias, interviewers were not informed about the expected outcomes of the study, or if the district was from an intervention or a control group.

Additionally, the interviewers used observation techniques to support and validate some the responses given by the households, namely those related to the effective use of LLIN. Interviewers explained the purpose of the study and obtained authorization and written informed consent; if the household member refused to participate, the questionnaire was applied to the nearest house.

### Variables

The questionnaire had questions related to the following quantitative and qualitative variables: (i) number of de facto people living in the household (people living in the same household for at least 6 months); (ii) presence or absence of campaign LLINs; (iii) number of campaign LLINs; (iv) use of campaign LLIN in the previous night and in the last four nights prior to the survey. All other existing LLINs (e.g., acquired from prenatal care or from campaigns prior to 2015, or from other source) were excluded from data collection during the interview and were considered as households without LLINs. The same approach was applied for those households that had campaign LLINs but slept under LLINs from another source; in this case was considered as owning campaign LLIN, but not sleeping under campaign LLIN. This was important to avoid information bias and effectively evaluate only the outcomes from the pilot.

### Households inclusion criteria

The following inclusion criteria were additionally used to select the households to be surveyed: (i) households from the selected districts, (ii) households living in the district since July 2015 (period of the beginning of the campaign preparations), (iii) interviewee with at least 18 years of age, regardless of gender.

### Outcomes of interest

The main outcomes are: (i) percentage of households with at least one LLIN in the intervention and control districts; (ii) percentage of population that slept under an LLIN the previous night (among the interviewees); (iii) percentage of LLIN owners that slept under an LLIN in the last four nights (among the interviewees); and (iv) percentage of households achieving universal coverage targets (one LLIN for every two persons).

### Statistical analysis

All data were introduced and analysed using SPSS version 23.0. Univariate and bivariate statistical analysis was performed. For quantitative variables descriptive statistics such as mean, median, and standard deviation [*SD*] were used, while absolute frequencies and percentage were calculated for qualitative variables. For universal coverage estimation, the number of LLINs available in each household was divided by the number of de facto members from the respective household. Values greater than or equal to 0.5 (meaning that one LLIN is for two persons) were considered as universal coverage target achievement. Subsequently the percentage of households that reached universal coverage was calculated.

In order to analyse associations between the district categories (intervention and control) and the main outcomes of the study (LLIN ownership, use, and universal coverage achievement), odds ratio (OR) and 95% confidence intervals (CI) were calculated. For all statistical procedures, a 0.05 significance level was adopted for rejecting the null hypothesis.

## Results

### Sample characteristics and number of campaign LLINs

There were 1547 households surveyed, of which 760 were in intervention and 787 in control districts. Both intervention districts have on average more LLINs per household (2.7, 95% CI 2.6–2.8) than control districts (2.3, 95% CI 2.2–2.4). Since the 95% mean confidence intervals between intervention and control districts do not overlap, a plausible mean LLIN difference between intervention and control districts can be considered (Table 1).

**Table 1:** Households composition (de facto members) and number of 2015 campaign LLINs in intervention and control districts

Districts	Intervention	Control
Surveyed households	760 (49.1%)	787 (50.9%)
	LLINs	LLINs
Mean	2.7	2.3
95% CI	2.6–2.8	2.2–2.4
Median	3.0	2.0
SD	1.3	1.7

Intervention (Gurue and Sussundenga); Control (Alto Molocue and Machaze)

### LLINs ownership, use, and universal coverage achievement in intervention and control districts

The percentage of household with LLIN was higher in the intervention districts than in the control districts. There was a significant association between households’ ownership of campaign LLIN and the delivery model [OR: 9.7, (95% CI 4.84–19.46)]. Although the use of LLIN in the previous night was above 80% in both the intervention and control districts, the LLIN use was higher in the intervention than in the control districts, and the difference observed was statistically significant [OR: 3.2; (95% CI 2.12–4.69)] (Table 2).

**Table 2:** LLIN household ownership, use and universal coverage 6 month after distribution in intervention and control districts

Districts	Households with LLINs				OR 95% CI	p value
	n	%	95% CI	Total		
Intervention	751	98.8	98.0–99.6	760	9.7 4.84–19.46	< 0.001
Control	705	89.6	87.5–91.7	787		
Total	1456	94.1	92.9–95.3	1547		
	LLIN use in previous night (slept under LLIN)					
Intervention	725	95.4	93.9–96.9	760	3.2 2.12–4.69	< 0.001
Control	683	86.8	84.4–89.2	787		
Total	1408	91.0	89.6–92.4	1547		
	LLIN use in the last 4 nights among LLIN owners					
Intervention	716	95.3	93.8–96.8	751	2.0 1.29–3.03	0.002
Control	643	91.2	89.1–93.3	705		
Total	1359	93.3	92.0–94.6	1456		
	Universal coverage target achievement (one LLIN for every two persons)					
Intervention	538	70.8	67.6–74.0	760	1.6 1.33–2.03	< 0.001
Control	469	59.6	56.2–63.0	787		
Total	1007	65.1	62.7–67.5	1547		

Intervention (Gurue and Sussundenga); Control (Alto Molocue and Machaze)

Amongst LLIN owners, the LLIN use in the last four nights (routine use) was also higher in the intervention than in control districts. There was a statistically significant association between routine use of LLINs and the delivery model [OR: 2.0; (95% CI 1.29–3.03)]. Of the 760 households surveyed in the intervention districts, 70.8% (95% CI 67.6–74.0) achieved the universal coverage target; of the 787 households surveyed in the control districts, 59.6% (95% CI 56.2–63.0) achieved the universal coverage target. There was a statistically significant association between percentage of households reaching universal coverage targets and the delivery model [OR: 1.6; (95% CI 1.33–2.03)] (Table 2).

## Discussion

This study shows that there were more households being covered with LLINs in the intervention districts when compared to control ones. The results also show more people using LLINs and better progress toward universal coverage target in the intervention districts. These results are consistent with other published studies and campaigns that used coupons to register households or sleeping spaces in preparation for LLIN campaign [3, 11–14].

The average LLINs per household increased when compared with what was observed in the 2015 nationwide survey (average of 1.5 LLINs per household) [8]. The intervention districts increased 1.2 LLINs per household; the control districts increased 0.8 LLINs per household.

**Fig. 1:**
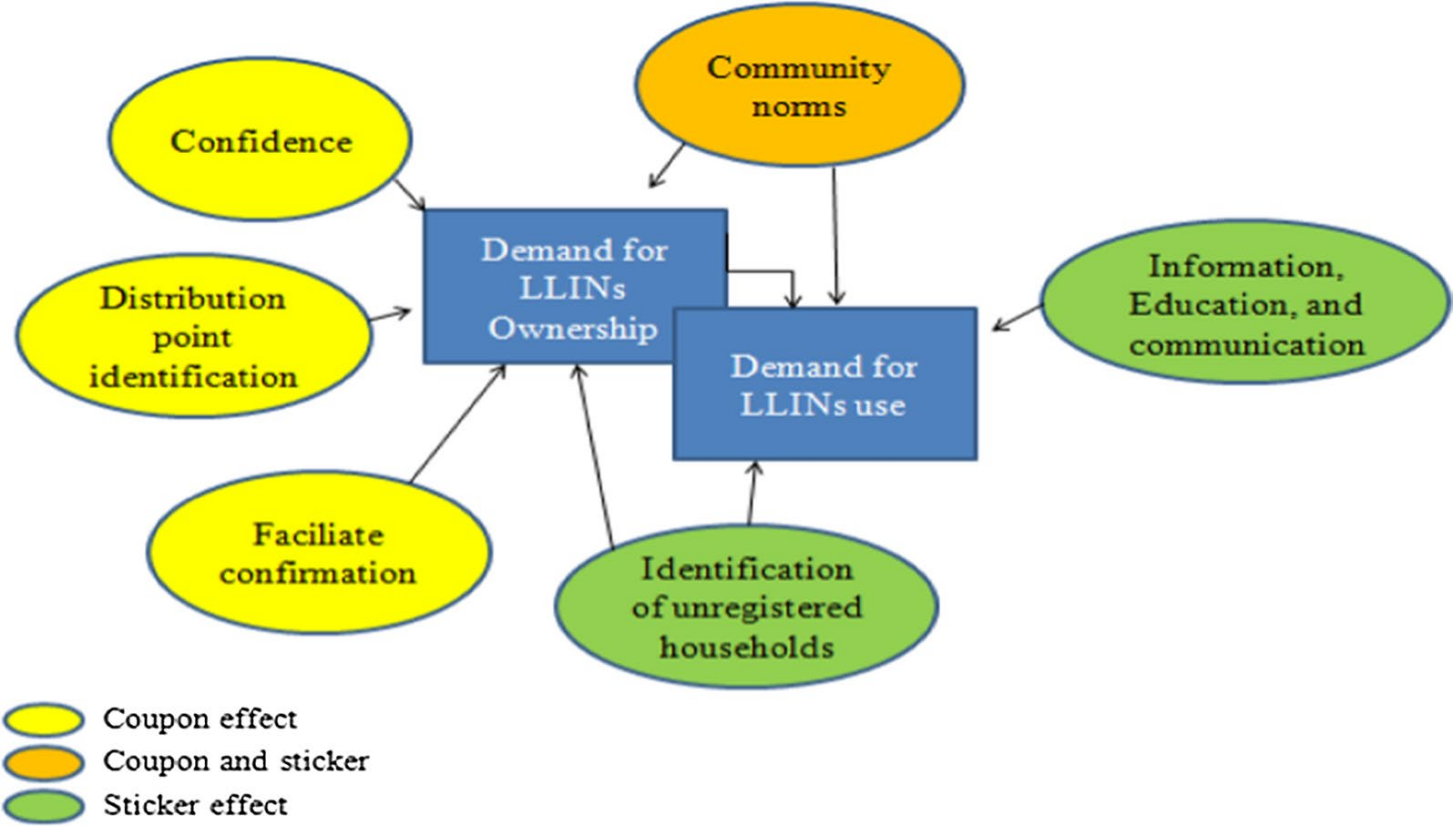
The coupon-sticker demand effect

The increased LLIN ownership among households in the intervention districts can be explained by what is herein referred to as the “coupon-sticker demand effect” [9]. The coupon effect is characterized by the following: (i) ensures the necessary confidence for the households that they will in fact receive LLINs; (ii) establishes community norms and give a meaning to the community, leading to the action of travelling to the place of distribution to exchange the coupon for LLINs; (iii) identifies the distribution point that households should go to in order to obtain LLINs; and (iv) facilitates confirmation that the household was registered, i.e., during LLINs distribution phase, the coupon is exchanged by LLINs. The sticker effect for LLIN demand is characterized by easy identification of unregistered households (i.e., households without a sticker), thereby ensuring that more households are registered and can benefit from LLINs. These two effects complement each other and create a positive gradient of demand behaviour, leading to more campaign LLIN access and ownership (Fig. 1).

Access to LLINs is one of the major determinants of their use [6]. LLINs are used by a high proportion of those who have access to them; therefore, the population sleeping under an LLIN closely tracks the proportion with access to an LLIN [6]. In spite of this, free distribution of LLINs has been shown to contribute to increased coverage and equity in their use [15]. With this in mind, the higher LLIN ownership coverage amongst households in the intervention districts (due to the “coupon-sticker demand effect”) might well lead to a higher chance of use rates. However, the effect of seasonality could also play a role in this high usage rate. The level of LLIN usage can be affected by factors such as temperature, humidity, season, and mosquito density [16], and reported usage levels might therefore be higher or lower depending on whether the survey were conducted in summer and the rainy season. Discomfort during LLIN use (primarily due to heat) might be experienced in summer, leading to low LLIN use rates. Heat was identified as a factor contributing to partial mosquito net use (i.e. use for part of the night, but not all) [17]. A review conducted by Pulford et al. [18] also reports discomfort, primarily due to heat, as the most widely identified reason why mosquito net owners chose not to use a mosquito net on one or more nights in the 17 survey-based studies included in the review. On the other hand, in the rainy season with high mosquito density, LLIN use might be higher.

Although the target of universal coverage is difficult to reach and sustain, this study shows that the new delivery model accelerates the pace toward this target. In fact, 71% of households in the intervention districts achieved universal coverage targets against 60% in the control districts. These results were higher than what was observed in nationwide 2011 and 2015 surveys, in which only 22.6 and 38.9% of households reached this target, respectively [7, 8]. The higher progression in the intervention districts might be explained by the “coupon-sticker demand effect”, the ascription formula used (one LLIN for every two persons), and the fact of no maximum number of LLINs per households being established, i.e., not capping. Another observation to remark upon is the fact that only LLINs distributed by the 2015 campaign were considered in this study. LLINs obtained from other sources, such as from prenatal care, were excluded, which may have underestimated the coverage. Finally, indicators such as ownership and usage rates should also be taken into account in addition to universal coverage for a better prediction of the impact of LLINs in interrupting malaria transmission.

## Conclusions

The universal coverage campaign piloted with the new delivery model, based on the use of coupons and stickers, has increased LLINs ownership, use, and progression for universal coverage targets in the community. The authors look forward to seeing the results of other countries’ experiences with these two core components during household registration phase. These encouraging results might well help National Malaria Control Programmes to improve the strategies for LLINs delivery model in campaigns, greatly helping to reduce the malaria burden across African countries.

## Abbreviations

CI: confidence interval
HH: household
LLIN/LLINs: long-lasting insecticidal nets
OR: odds ratio
SD: standard deviation
